# Galectin-1 promotes hepatocellular carcinoma and the combined therapeutic effect of OTX008 galectin-1 inhibitor and sorafenib in tumor cells

**DOI:** 10.1186/s13046-019-1402-x

**Published:** 2019-10-22

**Authors:** Zoe Leung, Frankie Chi Fat Ko, Sze Keong Tey, Ernest Man Lok Kwong, Xiaowen Mao, Bonnie Hei Man Liu, Angel Po Yee Ma, Yi Man Eva Fung, Chi-Ming Che, Danny Ka Ho Wong, Ching Lung Lai, Irene Oi-Lin Ng, Judy Wai Ping Yam

**Affiliations:** 1 Department of Pathology, The University of Hong Kong, Hong Kong, China; 2 Department of Chemistry, The University of Hong Kong, Hong Kong, China; 3 State Key Laboratory of Synthetic Chemistry, The University of Hong Kong, Hong Kong, China; 4 Department of Medicine, Queen Mary Hospital, The University of Hong Kong, Hong Kong, China; 5 State Key Laboratory of Liver Research (The University of Hong Kong), Hong Kong, China; 6 Department of Pathology, Block T, Queen Mary Hospital, Hong Kong, China

**Keywords:** Galectin-1, Hepatocellular carcinoma, miR-22, Therapeutics, OTX008

## Abstract

**Background:**

Galectins are beta-galactose specific binding proteins. In human cancers, including hepatocellular carcinoma (HCC), galectin-1 (Gal-1) is often found to be overexpressed. In order to combat the dismal diagnosis and death rates of HCC, gene silencing and targeted inhibition of Gal-1 was investigated for its improved therapeutic potential.

**Methods:**

Cellular and secretory Gal-1 levels were analyzed using HCC clinical samples. The study of Gal-1 was carried by both knockdown and overexpression approaches. The stable clones were tested by in vitro assays and in vivo experiments. Mass spectrometry was used to identify downstream targets of Gal-1. The upstream regulator of Gal-1, microRNA-22 (miR-22) was characterized by functional assays. The therapeutic effect of inhibiting Gal-1 was also analyzed*.*

**Results:**

Gal-1 overexpression was observed in HCC and correlated with aggressive clinicopathological features and poorer survival. The loss of Gal-1 resulted in hindered cell migration, invasion and anchorage independent growth. This was also observed in the animal models, in that when Gal-1 was knocked down, there were fewer lung metastases. Proteomic profiling of control and Gal-1 knockdown cells identified that the level of retention in endoplasmic reticulum 1 (RER1) was suppressed when Gal-1 level was reduced. The cell motility of Gal-1 knockdown cells was enhanced upon the rescue of RER1 expression. In HCC tissues, Gal-1 and RER1 expressions displayed a significant positive correlation. The upstream regulator of Gal-1, miR-22 was observed to be underexpressed in HCC tissues and negatively correlated with Gal-1. Silencing of miR-22 resulted in the upregulation of Gal-1 and enhanced cell growth, migration and invasion. However, such enhancement was abolished in cells treated with OTX008, an inhibitor of Gal-1. Combinational treatment of OTX008 and sorafenib significantly reduced tumor growth and size.

**Conclusions:**

Gal-1 overexpression was detected in HCC and this played a role in promoting tumorigenic processes and metastasis. The function of Gal-1 was found to be mediated through RER1. The correlations between miR-22, Gal-1 and RER1 expressions demonstrated the importance of miR-22 regulation on Gal-1/RER1 oncogenic activity. Lastly, the combinational treatment of OTX008 and sorafenib proved to be an improved therapeutic option compared to when administering sorafenib alone.

**Electronic supplementary material:**

The online version of this article (10.1186/s13046-019-1402-x) contains supplementary material, which is available to authorized users.

## Background

Hepatocellular carcinoma (HCC) is one of the top most occurring cancers and leading causes of cancer mortality in the world [1, 2]. The multistep, inflammation-associated progression of HCC is a major contributor to its development and reflects its aggressive nature. Exposure to its main risk factor, Hepatitis B virus (HBV), predisposes the liver to inflammation known as hepatitis. However, a sustained chronic inflammation of the liver leads to liver cirrhosis. The majority of HCC cases, approximately 80%, have an underlying association with cirrhosis. A combination of this long developmental process and asymptomatic nature of HCC consequently results in late detection and diagnosis of HCC, often at an advanced stage. Therapeutic options are therefore limited. The current treatments for advanced inoperable HCC include sorafenib and its derivative, regorafenib (Nexavar, Bayer HealthCare Pharmaceuticals - Onyx Pharmaceuticals). These multi-kinase oral inhibitors predominantly target the Raf/Mek/Erk pathway and as a result lead to the attenuation of various tumorigenic processes. For instance, sorafenib inhibits VEGFR, a vital mediator in the angiogenic process, and also cell cycle components p21 and p53 to impede cell cycle activities [3]. The efficacy of this drug is markedly more obvious in patients who have undergone liver transplantation, with an observed extended survival rate of approximately 14 months [4]. However, due to the complications involved in surgery, patients who have advanced HCC that cannot undergo surgery will often be prescribed sorafenib with undesirable responses. An increasing body of evidence has identified adverse effects of its treatment in patients including gastrointestinal issues, weight loss and vomiting [5]. With a median extended survival time of only approximately 3–5 months [6], the efficacy of sorafenib and/or other HCC treatments requires vast improvements.

Galectins are a family of B-galactose specific binding proteins, with a highly conserved carbohydrate recognition domain (CRD) present in all members of this family across a wide range of species. Galectin-1 (Gal-1), a 14.5 kDa prototypic galectin, is widely expressed in many tissue types. The single carbohydrate recognition domain of Gal-1 homo-dimerizes and allows Gal-1 to bind to various target glycoprotein ligands for its activity. Galectins in the normal liver physiology plays integral roles in the activation of cell surface receptors and result in galectin dimerization for cell-cell adhesion and binding of cell-matrix interactions. Gal-1 has been demonstrated to bind to extracellular matrix (ECM) components such as laminin, and that Gal-1 binding is dependent on the amount of substrate present for cell adhesion [7]. This highlights the natural adhesive characteristic of Gal-1.

The dysregulation of this Gal-1-carbohydrate interaction has, therefore, resulted in increased cell activity, adhesion and further downstream processes which in turn enhances tumor development. This oncogenic activity has been identified in many cancers including breast and gastric cancer [8, 9]. Furthermore, Gal-1 overexpression was found to be associated with enhanced cancer stem cell properties as identified in CD133+ lung cancer cells, where increased Gal-1 promoted oncogenesis [10]. Consequently, Gal-1 has been an attractive target for cancer therapeutics. Previous reports have identified the overexpression of Gal-1 in HCC to trigger epithelial-mesenchymal transition (EMT) and also allowing cells to become resistant to sorafenib through the PI3K/Akt signaling [11]. Therefore the presence of Gal-1 overexpression is undesirable.

In light of the extensive studies on Gal-1 overexpression in various tumors, its specific inhibitor, OTX008, has been developed and has completed the first phase of its clinical trial. Promising results from this trial has demonstrated the efficacy of OTX008 in reducing Gal-1 in patient serum levels, which was investigated in various types of solid tumors [12]. The need for this Gal-1 inhibitor implies the significance of understanding Gal-1 activity in solid tumors.

Over the years, increasing evidence supported the involvement of microRNAs (miRNA) in cancer progression. These 21–22 nucleotide long non-coding RNA molecules are capable of regulating gene expression through the silencing of their specific mRNA targets. Therefore, miRNAs are capable of suppressing and/or promoting tumors development. Previous studies have identified the level of miR-22 to be significantly lower in HCC tumor tissues [13, 14]. This underexpression resulted in suppressed Gal-1 mRNA expression and thus could be one of the reasons for Gal-1 overexpression in HCC.

In this study, we present the effects of reduced Gal-1 expression in HCC cells in diminishing HCC tumorigenesis. A negative correlation between miR-22 and Gal-1 was demonstrated by both the stable increase and decrease in miR-22 expression, which resulted in subsequent changes in Gal-1 activity in HCC cells. Furthermore, the treatment of sorafenib and OTX008 was tested for their combined inhibitory effects in attenuating tumor formation in animal models.

## Methods

### Clinical HCC samples

HCC paired patient samples were obtained upon surgical resection from Queen Mary Hospital, Hong Kong. The normal surrounding tissue and the tumor tissue from the same patient corresponds to the non-tumorous and tumorous counterpart, respectively. The cases used for RNA and tumor tissue analyses were selected at random, with no patient exclusion criteria. A total of 97 cases were subjected for qPCR analysis. A HCC tissue microarray consisting of 97 paired tumorous and non-tumorous cases was used for analyzing Gal-1 expression using immunohistochemistry. Blood serum samples including normal subjects, non-cirrhotic HBV patients, patients with HBV-associated cirrhosis and HCC were kindly prepared and provided by the Department of Surgery and the Department of Medicine, The University of Hong Kong. Gal-1 concentration in the serum was analyzed by Enzyme-linked immunosorbent assay.

### Cell culture and stable transfection reagents

The 293FT cell line, the MIHA immortalized normal liver cell line and the PLC/PRF/5 HCC cell lines were purchased from American Type Culture Collection (ATCC), USA. Human HCC cell lines BEL7402 and SMMC7221 were obtained from the Shanghai Institute of Cell Biology, Chinese Academy of Sciences, People’s Republic of China (PRC). The metastatic HCC cell line MHCC97L was provided by Fudan University, PRC. For in vivo bioluminescence imaging, MHCC97L was luciferase-labeled by lentiviral-based transduction. All cells were cultured in Dulbecco’s Modified Eagle Medium (DMEM) containing high glucose, L-glutamine, supplemented with 3.7 g/L sodium bicarbonate, 50 U/ml each of penicillin and streptomycin, and a final supplement of 10% heat-inactivated fetal bovine serum (FBS). To establish Gal-1 knockdown stable clones, shRNA against Gal-1 was transfected into 293FT cells using the FuGene® 6 transfection reagent (Promega) for the generation of viral particles. The infected cells were subjected to puromycin (Gibco®) selection for the establishment of stable shGal1 knockdown clones. For the stable expression of miR-22 into HCC cells, lentiviral particles for the miR-22 precursor (GeneCopoeia, Rockville, MD, USA) was transfected into various HCC cell lines for the increase in miR-22 expression. Conversely, for the reduction of miR-22, lentiviral particles inhibitor for miR-22 (GeneCopoeia) was transfected with GeneCopoeia Lenti-Pac™ HIV expression packaging system into 293FT cells before viral transduction into HCC cell lines. The pCMV3-RER1 expression plasmid (Sino Biological) was transfected into Gal-1 knockdown cells using Lipofectamine 2000 Transfection Reagent (Invitrogen).

### Animal model

For subcutaneous injection of cells, 1 × 10^6^ cells resuspended in 100 μl PBS were injected into the back of 4–5 weeks old male BALB/c nude mice. Tumor growth was monitored weekly by measuring the length and width of the tumor. The tumor volume was calculated using the formula: 0.5 × Length × Width^2^. For orthotopic implantation, 3 × 10^6^ cells resuspended in 100 μl PBS, were subcutaneously injected into the flank of mice. Tumors were left to grow for up to three weeks before mice were sacrificed. For tumor implantation, the tumor seed was cut into approximately 1 mm^3^ cubes and implanted into the liver of 6-week old mice. After administering anesthesia to the mice, the liver of the mice was exposed and mechanically injured with a surgical needle. The tumor cube was implanted into the incision and then secured by suture. Tumor growth in the liver was monitored weekly for up to 6 weeks via bioluminescent imaging. This was carried out by anaesthetizing the animal and then injecting it with D-luciferin (Xenogen, Hopkinton, MA, USA), which is required for bioluminescent signaling. Images were captured and analyzed by the IVIS 100 Imaging System (Xenogen). At the end of the experiment, in order to observe for any metastatic events, the lungs had to be removed from the animal prior to bioluminescent imaging.

For drug administration, tumors were allowed to reach approximately 5 × 5 mm in size before drug treatment. The mice were randomized into 4 groups, namely control (vehicle), OTX (5 mg of OTX008/3 days, administered intraperitoneally), sorafenib (30 mg/kg/day, administered orally) and OTX + sorafenib.

### Immunohistochemical (IHC) analysis

IHC analysis was carried out on fixed, paraffin-embedded blocks of patient samples and excised animal organs. The LGALS1 antibody (Abcam, ab138513, 1:250 dilution) was used for IHC staining. The detailed protocol is described in our previous study [15]. Briefly, paraffin-embedded tissues were cut to 5-um thick before dewaxing in xylene. Samples were then rehydrated in decreasing alcohol gradients and then water. Further antigen retrieval and blocking of endogenous peroxidase processes were carried out before primary antibody incubation. Excess primary antibody was removed and washed thoroughly in TBST before secondary antibody was added to the slide and incubated for 30 min. Slides were then counterstained with hematoxylin and eosin (H&E). Histological analysis was performed with Aperio ScanScope CS system camera by pathologists.

### Mass spectrometry

Cell lysate digest were desalted and loaded onto the self-packed column with autosampler for LC-MS/MS analysis. One μg of peptides was loaded onto the column with 100% A (0.1% formic acid) and then eluted for 180 mins with a gradient from 5% B to 27% B (0.1% formic acid in 80% ACN) at a flow rate of 150 ηL/min with a nano-flow UPLC (Easy-nLC 1200). During the gradient, the MS (Orbitrap Fusion Tribrid mass spectrometer, Thermo Fisher Scientific) was operated at an automatic mode in which it switched between a survey full MS scan and tandem MS/MS CID scans for 3 s on the most abundant peaks. The generated results’ raw files were searched with MaxQuant search engine against the Uniprot human database (70,902 entries). The search was specified to trypsin digestion, methionine oxidation as dynamic modification, and carbamidomethylation of cysteine as fixed modification. Heat map was used to reveal the overall differentially expressed proteins in shCtrl and shGal1 cells. A ratio less than 0.5 represents ≥2-fold downregulation and a ratio larger than 2 represents ≥2-fold upregulation in protein expression in shGal1 cells when compared to shCtl cells.

### Statistical analysis

Statistical analysis of patients and clinical data were analyzed by the Fisher’s exact test with IBM SPSS statistics 20. All in vitro functional assays were analyzed by the student *t* test for statistical significance using GraphPad Prism 6 (San Diego, CA, USA). A *P*-value of less than 0.05 is considered statistically significant.

## Results

### Elevated Gal-1 expression in HCC tissues correlates to the poor prognosis and aggressive metastatic features

**Fig. 1:**
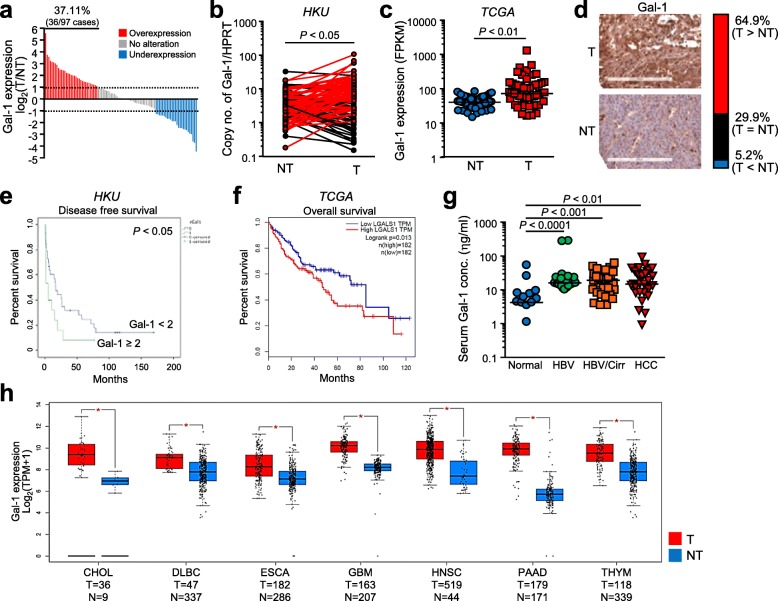
Overexpression of Gal-1 was associated with poor clinical outcome in HCC patients. **a.** Gal-1 overexpression in 37.11% (36/97) of paired HCC cases comparing tumorous (T) and non-tumorous (NT) tissues. **b.** HKU cohort consisted of a significant overexpression of Gal-1 (*n* = 97). **c.** This trend was similarly observed in The Cancer Genome Atlas (TCGA) cohort for liver cancer (*n* = 50). **d.** Intense Gal-1 staining in tumorous tissues compared to non-tumorous tissues found in 64.9% of cases through IHC analysis. **e.** Clinicopathological analysis of patients with Gal-1 overexpression indicated poorer disease-free survival. **f.** Based on TCGA database, Gal-1 overexpression was found to be associated with poorer overall survival. **g.** The level of Gal-1 secretory analyzed by ELISA in patient blood serum revealed the increased Gal-1 levels in patients with HBV, cirrhosis and HCC. HBV: hepatitis B virus; Cirr: cirrhosis; HCC: hepatocellular carcinoma. **h.** Identification of Gal-1 overexpression observed in other cancers from TCGA database comparing tumor and non-tumor counterparts. CHOL: cholangioma; DLBCL: diffuse large B-cell carcinoma; ESCA: esophageal carcinoma; GBM: glioblastoma; HNSC: head and neck squamous cell carcinoma; PAAD: pancreas adenocarcinoma; THYM: thymoma. *P < 0.05 is considered to be statistically significant

Tumorous samples were compared to their non-tumorous counterparts for the evaluation of Gal-1 overexpression in clinical tissues. From these experiments, in 37.11% (36/97) of the cases, a 2-fold Gal-1 overexpression was observed in tumor tissues (*P* < 0.05) (Fig. 1a & b). A similar trend was observed in the TCGA cohort of liver cancer with significant Gal-1 overexpression in tumor samples (*P* < 0.01) (Fig. 1c). Consistently, IHC staining using the tissue microarray (*N* = 97) revealed a higher Gal-1 expression in tumorous tissues than in the corresponding non-tumorous tissues. Overexpression of Gal-1 was determined by the positive Gal-1 staining in the tumorous tissue compared to the adjacent non-tumorous tissue. The Gal-1 overexpression, underexpression and unchanged expression were observed in 64.9, 5.2 and 29.9% of the cases, respectively (Fig. 1d). Furthermore, to examine whether overexpression of Gal-1 is correlated to metastatic potential, statistical analysis revealed that Gal-1 overexpression correlated with the absence of tumor encapsulation (*P* < 0.01) and also the presence of microsatellites (*P* < 0.05) (Table 1). Moreover, Gal-1 overexpression was found to be correlated with poorer disease free survival (*P* < 0.05) (Fig. 1e). The consequence of increased Gal-1 level was also reinforced in the poorer overall patient survival, as supported with the data from the TCGA database of liver cancer (Fig. 1f).

**Table 1:** Correlation of galectin-1 with the histopathological parameters of HCC patients

Histopathological parameters	T/NT < 2 (without Gal-1 overexpression)	T/NT ≥ 2 (with Gal-1 overexpression)	*P*-value
Sex			
Male	47	23	0.794
Female	13	8	
Cirrhotic liver			
Cirrhosis	18	6	0.402
NT & hepatitis	22	14	
HBsAg			
Positive	38	16	0.712
Negative	6	4	
Cellular differentiation			
Poor	22	8	0.130
Differentiated	18	12	
Tumor size			
> 5 cm	26	15	0.560
≤ 5 cm	14	5	
Tumor encapsulation			
Absent	21	16	0.008*
Present	27	4	
Venous invasion			
Present	27	19	0.270
Absent	29	12	
Microsatellite			
Present	17	15	0.032*
Absent	25	6	
Direct liver invasion			
Present	11	7	0.551
Absent	23	10	
Tumor nodule			
*N* ≥ 2	10	7	0.547
*N* = 1	29	13	

*T* Tumorous, *NT* non-tumorous, *N* number of tumor nodule, *HBsAg* Hepatitis B surface antigen, *P* < 0.05 is regarded as statistically significant

*P < 0.05 is regarded as statistically significant

Comparatively, the level of Gal-1 expression was analyzed in tumorous and non-tumorous counterparts in various cancers (Fig. 1h). The significant overexpression of Gal-1 in these cancers including cholangiocarcinoma (CHOL), diffuse large B-cell carcinoma (DLBCL), esophageal carcinoma (ESCA), glioblastoma (GBM), head and neck squamous cell carcinoma (HNSC), pancreas adenocarcinoma (PAAD) and in thymoma (THYM). This, therefore, reiterates the importance of understanding Gal-1 expression in cancer and the clinical implications of its overexpression.

### Serum Gal-1 level is correlated with HBV and cirrhotic conditions of the liver

As Gal-1 is a secretory protein, we further investigated whether Gal-1 levels in blood sera would vary in HCC patients compared to normal individuals. In 177 HCC patient samples, statistical analysis revealed a significant increase in Gal-1 levels in an inflammation-associated manner when compared to normal individuals (Fig. 1g). As HCC coincides with HBV infection and inflammation, the increased level of Gal-1 in patients with HBV infection (*P* < 0.0001) may suggest that Gal-1 plays a role in the initiation of HCC development. Consistently, the level of Gal-1 is also significantly increased in patients with both HBV infection and underlying cirrhosis (*P* < 0.001) and HCC patients, compared to normal individuals (*P* < 0.01). As HCC develops in enhanced inflammatory conditions, this correlation between high Gal-1 serum levels and inflammation could suggest that Gal-1 levels increase depending on the severity of liver inflammation and damage.

### Abolishing Gal-1 reduces anchorage independent growth and HCC cell motility

**Fig. 2:**
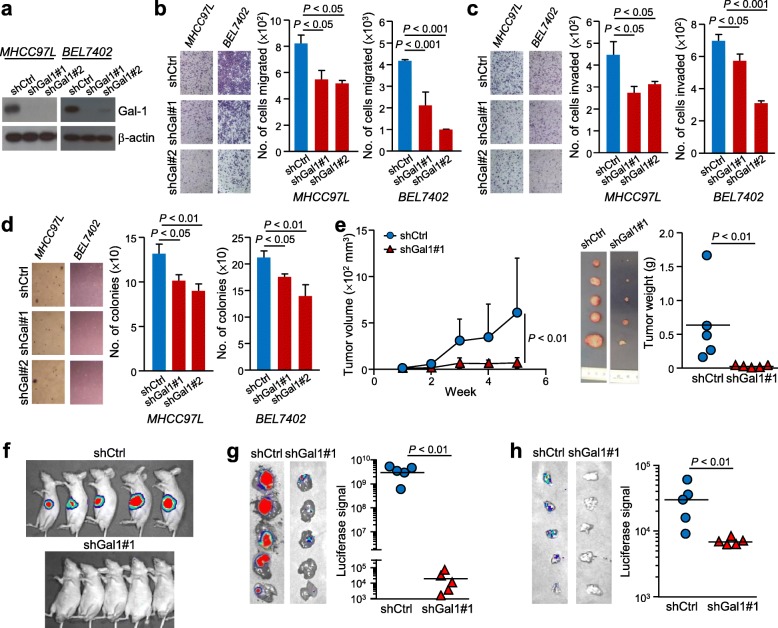
Knockdown of Gal-1 diminished HCC cell aggressiveness in vitro and in vivo. **a.** Knockdown of Gal-1 expression verified by western blot in both knockdown clones in two cell lines, MHCC97L and BEL7402. **b.** Migration assays using shGal1 cells reduced the number of cells migrated in both cells lines. **c.** Invasion assays with knocked down Gal-1 in cells resulted in fewer number of cells invaded in both cell lines. **d.** Soft agar assay results show fewer colonies formed when Gal-1 was knocked down. Representative fields for each group are also presented. **e.** Subcutaneous injection of shGal1 cells in BALB/c nude mice reduced in vivo tumor growth compared to shCtrl cells, indicated by the reduced bioluminescent signal and tumor volume (*n* = 5). **f.** Excised tumors are significantly smaller in tumor weight when Gal-1 was knocked down compared to shCtrl. **g.** Orthotopic implantation of tumors into liver of nude mice revealed lower bioluminescent signals. **h.** The reduced luciferase signal in shGal1 tumors compared to shCtrl-derived tumors represent reduced metastatic events when Gal-1 has been knocked down. Results are expressed as mean +/− SD. *P* < 0.05 is considered statistically significant

The success of knocked down Gal-1 expression in MHCC97L and BEL7402 cells was verified by western blot and qPCR (Fig. 2a). As the migratory abilities of cells are associated with metastatic ability, we investigated the consequence of Gal-1 knockdown in HCC cell migration and invasion. Analysis revealed that knocking down Gal-1 diminished the number of cells migrated (*P* < 0.05) and invaded (*P* < 0.001) (Fig. 2b & c). In the soft agar assay, when compared to control cells (shCtrl), Gal-1 knockdown clones (shGal1#1 and #2) displayed a significant reduction in the number of colonies formed (*P* < 0.05 and *P* < 0.01, respectively) (Fig. 2d). Additionally, the level of secretory Gal-1 was also significantly reduced when Gal-1 was stably knocked down, suggesting that the level of Gal-1 secreted can also be reduced (*P* < 0.01) (Additional file 1: Figure S1a). The addition of recombinant Gal-1 protein was found to replenish the reduction of Gal-1 in these cells, as evidenced in the increased number of cells migrated (*P* < 0.01) and invaded (*P* < 0.01) (Additional file 1: Figure S1b & c).

The importance of Gal-1 in HCC cells was also reinforced by an overexpression experiment with Gal-1 overexpressed in PLC/PRF/5 cells (Additional file 1: Figure S2a). This increased Gal-1 level was reflected in the increased ability of the cells to form colonies in a soft agar assay (*P* < 0.0.5) (Additional file 1: Figure S2b), and in the number of cells migrated (*P* < 0.05) and invaded (*P* < 0.05) (Additional file 1: Figure S2c & d).

### Knocking down Gal-1 reduces the ability of in vivo tumorigenicity and metastatic potential

The control and one Gal-1 knockdown clone of the metastatic MHCC97L was selected to proceed with in vivo studies. The MHCC97L was luciferase-labeled which enabled the analysis of tumors and metastasis in animals. At the end of the subcutaneous injection assay, the growth curve revealed the tumor volume to be significantly smaller when Gal-1 level was knocked down (*P* < 0.01), with tumor weight also being significantly reduced when compared to the control (*P* < 0.01) (Fig. 2e). In an orthotopic implantation experiment, the bioluminescent signal of animals with the implanted tumor derived from MHCC97L shCtrl cells was more prominent than animals implanted with shGal1 tumor at the end of the experiment (Fig. 2f). Consistently, the excised liver exerted a lower luciferase signal in the shGal1 tumor group compared to the control shCtrl group (*P* < 0.01) (Fig. 2g). The reduced distant metastatic potential was reflected in the lungs, which emitted less bioluminescent signal in animals implanted with shGal1 tumor when compared to those implanted with shCtrl tumor (*P* < 0.01) (Fig. 2h) and thus suggesting that Gal-1 in HCC enhances the ability of tumor cells to metastasize to the lungs.

### miR-22 negatively regulates Gal-1 expression in HCC

**Fig. 3:**
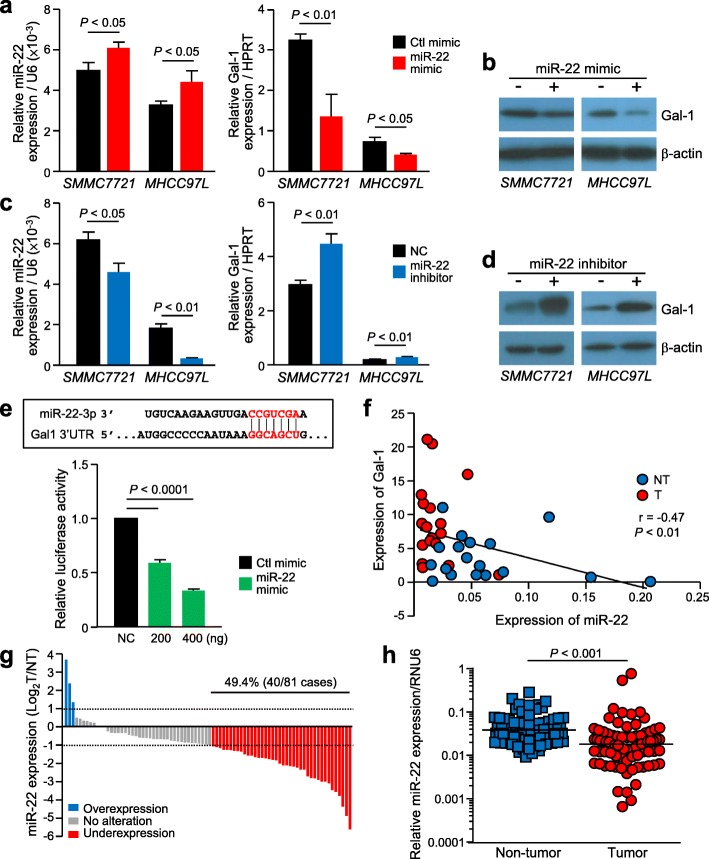
Negative regulation of Gal-1 by miR-22 in HCC. **a.** qPCR analysis revealed upregulation of miR-22 and reduced level of Gal-1 in cells transfected with miR-22 mimic. **b.** Reduction of Gal-1 was also detected by western blotting. **c.** Reduction of miR-22 expression by miR-22 inhibitor and the subsequent Gal-1 increased expression by qPCR and western blot **(d)**. **e.** Verification of direct correlation of Gal-1 and miR-22 through the putative binding site of miR-22 and Gal-1 3’UTR mRNA. Dual luciferase assay revealed the reduction in luciferase activity when miR-22 mimic was co-treated in these cells, compared to negative control (NC). **f.** A negative correlation was identified in HCC clinical samples when comparing Gal-1 and miR-22 expression in non-tumorous (NT) and tumorous (T) tissues of HCC patients. **g.** miR-22 expression was analyzed in 81 paired HCC cases. Comparing tumor and non-tumor samples, miR-22 was found to be underexpressed in 49.4% of the cases (40/81). miR-22 expression was normalized with RNU6, a housekeeping gene for data normalization. **h.** The overall expression of miR-22 was reduced in tumorous tissues when compared to non-tumorous tissues. *P* < 0.05 is considered statistically significant

In silico analysis using TargetScanHuman 7.0 revealed that miR-22 was the only miRNA that regulates Gal-1. The negative association between Gal-1 and miR-22 was firstly observed by transient transfection of miR-22 in HCC cells. The enhanced level of miR-22 in cells was determined by qPCR, and the corresponding reduced Gal-1 level was analyzed by both qPCR and western blot analysis (Fig. 3a & b). Conversely, the reduced transient expression of miR-22 by a miR-22 inhibitor resulted in increased Gal-1 levels (Fig. 3c & d). The direct regulation of this relationship was demonstrated in a dual luciferase reporter assay with the addition of varying concentrations of miR-22 mimic. A significant reduction in luciferase activity was observed with the addition of miR-22 mimic compared to the negative control (NC), which verified the direct negative relationship between the expression levels of miR-22 and Gal-1 (*P* < 0.0001) (Fig. 3e). The negative correlation was also seen in HCC cases in which both Gal-1 and miR-22 levels were deregulated (Fig. 3f). To reinforce these findings, qPCR analysis revealed significant miR-22 underexpression in 49.4% of the cases in tumor samples when compared to the paired non-tumorous tissues (*P* < 0.001) (Fig. 3g & h). However, miR-22 underexpression was not associated with any clinicopathological parameters (Additional file 1: Table S1).

### OTX008 inhibitor significantly abrogates HCC cell aggressiveness induced by dysregulated miR-22

**Fig. 4:**
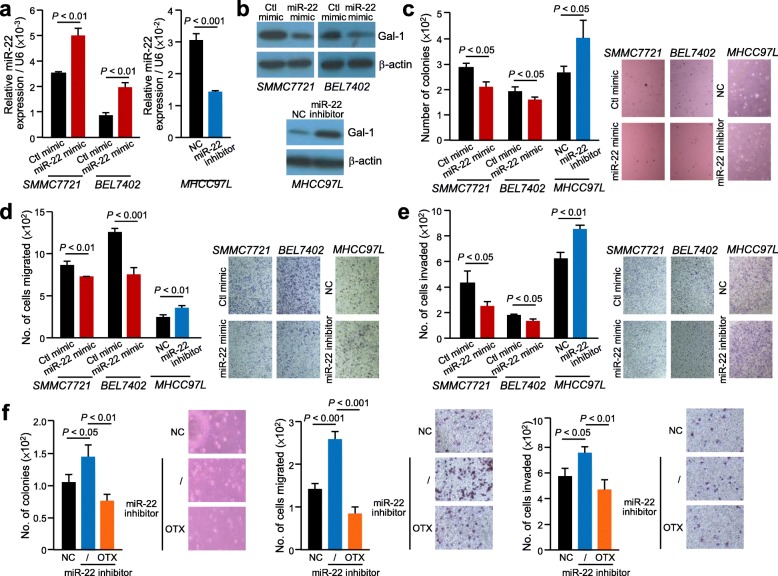
Inhibition of miR-22 enhanced HCC cell aggressiveness and such enhancement was abolished by treatment with inhibitor of Gal-1. qPCR (**a**) and western blotting (**b**) revealed increase of miR-22 and decreased Gal-1 protein expression in stable miR-22 mimic cells, whereas transfection of miR-22 inhibitor in cells resulted in enhanced Gal-1 protein level. **c.** Anchorage independent growth assays of stable miR-22 mimic and miR-22 inhibitor cells. Stable miR-22 mimic and miR-22 inhibitor cells were subjected to migration (**d**) and (**e**) invasion assays. **f.** The increased number of colonies formed and increase cell migration and invasion was observed in miR-22 inhibitor stable cells compared to control cells was diminished when miR-22 inhibitor cells treated with OTX008 (OTX). Results are expressed as mean +/− SD. *P* < 0.05 is considered statistically significant

Stable expression of miR-22 mimic and inhibitor resulted in reduced and elevated levels of Gal-1, respectively (Fig. 4a & b). In functional assays, when altering the levels of miR-22 in SMMC7221, BEL7402 and MHCC97L cells, the ability for anchorage independent growth was significantly reduced in the miR-22 mimic cells (*P* < 0.05), whereas the number of colonies formed significantly increased when miR-22 expression was inhibited in MHCC97L cells (*P* < 0.05) (Fig. 4c). Similarly, subjecting these cells to migration and invasion assays showed a similar trend, where inverse level of miR-22 affected the number of cells migrated and invaded (Fig. 4d & e).

Treatment of OTX008 on stable MHCC97L miR-22 inhibitor cells revealed that the Gal-1 activity significantly decreased post treatment. In vitro assays revealed that the HCC cell aggressiveness was significantly inhibited by OTX008 in these cells (Fig. 4f).

### Gal-1 promotes HCC cell migration and invasion through the upregulation of RER1

**Fig. 5:**
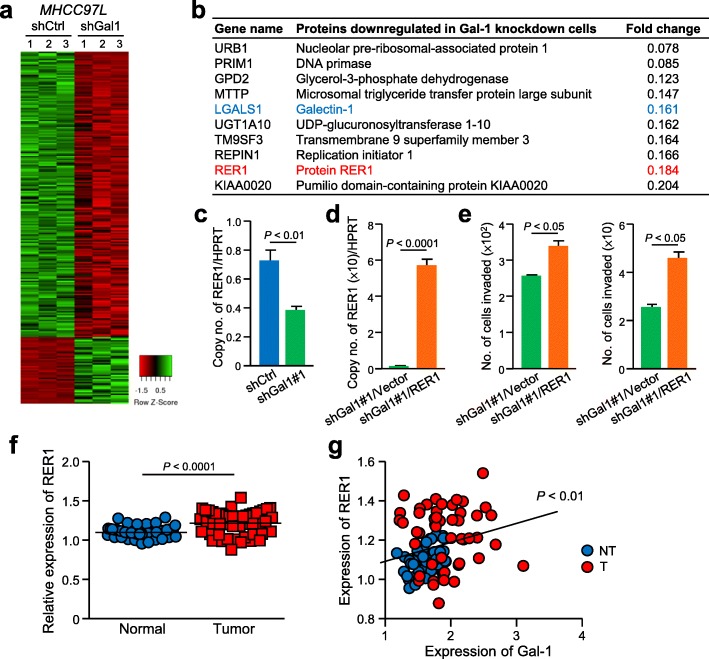
Gal1-mediated upregulation of RER1 promotes HCC migration and invasion. **a.** Heat map revealed expressions of protein candidates with at least 2-fold difference between non-target control (shCtrl) and Gal-1 knockdown cells (shGal1#1). **b.** Top ten listed genes downregulated in Gal-1 knockdown cells when compared to control cells. A ratio with less than 0.5 represents a more than 2-fold downregulation in shGal1 cells. **c.** Quantitative PCR analysis of RER1 expression in the control and Gal-1 knockdown cells. **d.** RER1 expression was rescued in Gal-1 knockdown cells by transfecting an expression vector of RER1 into shGal1#1. Quantitative PCR revealed the upregulation of RER1 in Gal-1 knockdown cells. **e.** Migration (*left*) and invasion (*right*) assays were performed using RER1-transfected Gal-1 knockdown cells. **f.** Expression of RER1 in 50 cases of paired HCC tumorous and non-tumorous tissues of TCGA database was compared. **g.** A positive correlation between Gal-1 and RER1 expressions was found in HCC cases obtained from TCGA database. *P* < 0.05 is considered statistically significant

To understand the molecular basis underlying the role of Gal-1 in HCC, mass spectrometry was performed to compare the expression profiles between MHCC97L shCtrl control and shGal1 knockdown cells. Distinct differentially expressed proteins with at least 2-fold difference were found in control and Gal-1 knockdown cells (Fig. 5a). Among the top listed downregulated proteins in shGal1 cells, RER1 which is a transmembrane protein localized at the Golgi apparatus was selected for further analysis after validation (Fig. 5b). The downregulation of RER1 in Gal1 knockdown cells was confirmed by quantitative PCR (Fig. 5c). To elucidate whether the activity of Gal-1 is mediated through RER1, RER1 expression was rescued in Gal-1 knockdown cells (Fig. 5d) and the RER1 overexpressed cells were analyzed for their migratory and invasive potentials. The results showed that both the migration and invasiveness of Gal-1 knockdown cells were significantly elevated upon the rescue of RER1 expression (*P* < 0.05) (Fig. 5e). Analysis of the HCC cases obtained from the TCGA database resulted in the overexpression of RER1 in HCC tissues when compared to paired non-tumorous tissues (Fig. 5f) and a significant positive correlation between Gal-1 and RER1 expressions (*P* < 0.01) (Fig. 5g).

### Combined treatment of OTX008 and sorafenib significantly reduces HCC tumorigenesis

**Fig. 6:**
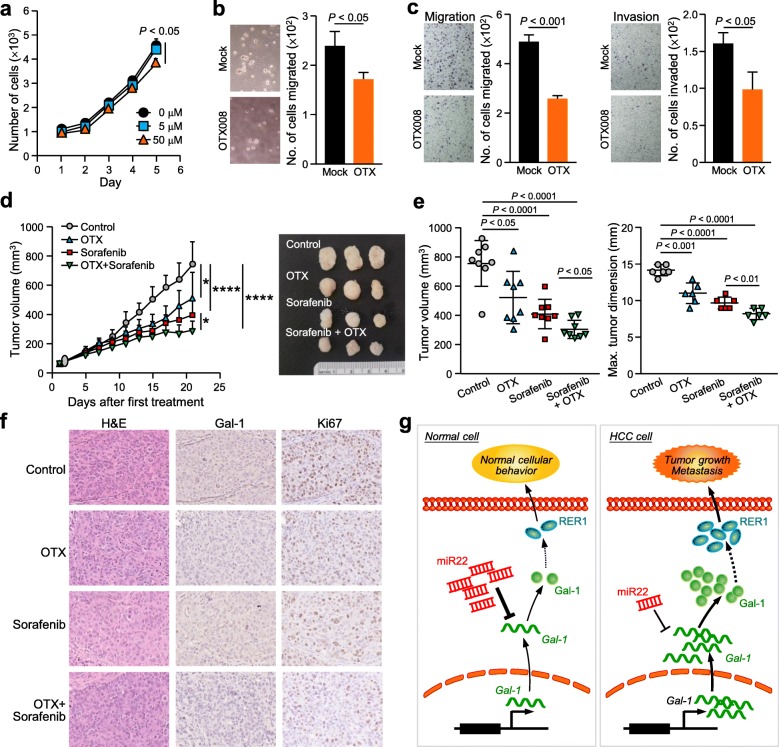
Targeted Gal-1 inhibition by OTX008 reduces oncogenic properties of Gal-1. **a.** A dosage of 50 μM OTX008 (OTX) was sufficient to inhibit MHCC97L cell proliferation rate. **b.** The same dosage was applied to MHCC97L cells to significantly reduce the number of colonies formed in a soft agar assay. **c.** The migratory and invasive abilities were reduced in MHCC97L cells after treatment. **d.** Tumor growth was monitored and significantly showed the inhibition of growth when mice were treated with sorafenib, OTX008 and the combined treatment of OTX008 and sorafenib, compared to the vehicle control. **e.** Combined treatment of sorafenib and OTX008 significantly attenuated end tumor volume and dimension. **f.** Representative images showing H&E and immunohistochemical staining of Gal-1 and Ki67 on tumors of different experimental groups are shown. **g.** Summary of the signaling of Gal-1 in HCC. *P < 0.05, ****P < 0.0001, P < 0.05 is considered statistically significant

The OTX008 dosage for optimum inhibition in HCC cells was determined by cell proliferation rate. MHCC97L cells was significantly inhibited when treated with 50 μM of OTX008 (*P* < 0.05), compared to 5 μM (Fig. 6a). This dosage also resulted in a significant reduction in the number of colonies formed (*P* < 0.05) (Fig. 6b), the number of cells migrated (*P* < 0.001) and the number of cells invaded (*P* < 0.05) in their respective assays (Fig. 6c). The animals subjected to subcutaneous injection of MHCC97L cells were randomly divided into 4 groups: 1) control, 2) OTX008 alone, 3) sorafenib alone and 4) combined OTX008 and sorafenib. Figure 6d shows the significant reduction in tumor volume compared to the control when administered with OTX008 alone (*P* < 0.05), sorafenib alone (*P* < 0.0001) and the combined treatment (*P* < 0.0001). The results also showed that the combination of OTX008 and sorafenib exerted the most potent effect in inhibiting tumor formation. Consistently, the tumor volume and dimension were significantly reduced between the different treatment groups (Fig. 6e). IHC analysis revealed that Gal-1 expression was lower in tumors treated with OTX008 and Ki67 positive cells were decreased in all treatment groups (Fig. 6f).

To summarize, the importance of Gal-1 in cells has been emphasized for its role in various cell activities including cell growth, motility and proliferation. In a normal cellular environment, miR-22 and Gal-1 levels are maintained at a regular level in order to achieve normal cell activity. However, under tumorigenic conditions, reduced miR-22 expression and the aberrant *LSGAL1* resulted in an increased level of Gal-1 protein leading to Gal-1/RER1-associated oncogenic processes (Fig. 6g).

## Discussions

With HCC consistently remaining as one of the top leading causes of cancer deaths worldwide, it has been integral in providing an insight into its complex development. Furthermore, the existing standard chemotherapy and patient care for advanced HCC patients has been lacking and thus reinforces the importance of improved treatment options.

In this study, we demonstrated the consequences of Gal-1 overexpression in HCC and the unfavorable effects this has on HCC cell activity. Given that the Gal-1 levels are elevated, as observed in various sources of HCC clinical samples, this implies that Gal-1 is associated with HCC metastasis in patients. Furthermore, the progressively increasing Gal-1 levels in blood sera of patients, who have been diagnosed with liver inflammation, could imply the oncogenic ability in initiating and promoting HCC development. This is supported by the elevated levels of Gal-1 in HBV-infected patients, patients with cirrhosis and HCC patients. Moreover, the metastatic ability of Gal-1 was clearly demonstrated by the animal model which revealed fewer lung metastases when Gal-1 levels were reduced.

Next, the regulation of Gal-1 under the activity of miR-22 was explored and this clear negative correlation could possibly explain the overexpression of Gal-1 in HCC patients. The significance of miR-22 in cancer has been previously reported, with its increased activity associated with suppression in tumor growth and metastasis. In a breast cancer model, through its direct interaction with its mRNA targets CDK6, SIRT1 and Sp1, which are genes involved in senescence, miR-22 has been able to suppress tumor growth [16]. In this study, although we have yet to explore the mechanism behind this negative correlation, this data could be an insight into how dysregulated miR-22 regulates Gal-1 in promoting HCC tumorigenesis. Various factors in the tumor microenvironment may cause the aberrant miR-22 expression in HCC, for instance, hypoxia. This oxygen-deprived condition has been observed in a vast amount of studies which is commonly found in solid tumors. This phenomenon occurs in fast growing solid tumors which result in the rapid depletion of oxygen for tumor cells. Interestingly, the activated hypoxia pathway enables tumor cells to adapt to these conditions and drive malignant tumor cell growth [17].

In one study, miR-22 was demonstrated to attenuate the hypoxia master regulator protein, HIF1α, whereas Gal-1 expression was enhanced under hypoxia [18]. Previous studies have shown Gal-1 levels to be enhanced under hypoxic conditions as Gal-1 has long been identified as a hypoxia-responsive gene [19]. Therefore, as Gal-1 can function intra- and extracellularly, a potential regulatory mechanism between miR-22 and Gal-1 could be explained by external factors such as the tumor microenvironment. The interaction between miR-22, HIF1α and Gal-1 could, therefore, be an interesting approach in understanding the upstream regulation of Gal-1 and miR-22 and also downstream oncogenic pathways between HIF1α and Gal-1.

By comparing the proteomic profiles of MHCC97L non-target control and Gal-1 knockdown cells, RER1 was found to be downregulated when Gal-1 level was reduced. Re-expression of RER1 in Gal-1 knockdown cells restored the migratory and invasive potentials of cells. RER1 has not been well characterized for its role in human cancers. The functional effect of RER1 has only been reported in human cancers in terms of its capacity to promote epithelial-mesenchymal-transition, stemness of cancer stem cells, tumorigenesis and metastasis in pancreatic cancer [20]. A recent study reported RER1 as one of the newly identified reference genes for quantifying cancer-related gene expression level [21]. The reference genes should theoretically be stably expressed and less likely to be affected by pathological conditions. Yet, RER1 has been found to be upregulated in pancreatic cancer [20] and also in liver cancer in our findings. However, the mechanism leading to the overexpression of RER1 in tumorous tissues remains unclear. RER1 has been shown to be upregulated by hypoxia-inducible factor 1α (HIF1α) [20]. Intriguingly, Gal-1 has been reported to be the downstream effector of HIF1α in clear cell renal cell carcinoma [18]. Together with our findings that RER1 expression was reduced in Gal-1 knockdown cells, it is postulated that HIF1α upregulates RER1 through Gal-1 which deserves further investigation.

With tumors being heterogenic in nature, it is important for drugs and inhibitors to be able to inhibit their targets specifically within this complex network. The ability of sorafenib in targeting a wide range of kinases has led to the development of Regorafenib. Regorafenib has been effective for patients who no longer have any therapeutic response to sorafenib. Sorafenib resistance has been a major drawback in this therapeutic drug and moreover, the various adverse effects include nausea, gastrointestinal problems and even hypertension, highlights the urgent need for HCC treatment improvement. Although Regorafenib has been found to extend patient survival to 10 months when compared to placebo [22], it still remains as the second-line therapy to sorafenib as they target similar kinases. The need for specific inhibitors is required for targeting different genes and signaling pathways involved in HCC tumorigenesis.

The treatment of OTX008 has proven to be successful in inhibiting Gal-1 activity in HCC cells. This promising inhibitor in treating patients with elevated Gal-1 levels has so far shown to significantly reduce Gal-1 serum levels in a clinical trial study [12] and from our study, we have also demonstrated the efficiency in Gal-1 activity and inhibiting various cancer cell related processes. OTX008 is specific in binding to one β-sheet of Gal-1, which results in its proteosomal degradation, albeit the exact mechanism is yet to be fully explored [23].

At the optimal concentration of OTX008, enhanced Gal-1 levels in tumors can be reduced as no functional protein is present and investigating its inactivity could potentially hinder HCC tumorigenesis. Since the first phase of the OTX008 clinical trial completed in May 2013, to our knowledge there has so far been no follow-up studies for its subsequent phases. However, this does not diminish the significance of OTX008 in cancer research. For instance, in this study, we showed that the oncogenic ability of Gal-1 in HCC cell activity can be attenuated by the treatment of OTX008. We further demonstrated the efficacy of the inhibitor with sorafenib as a combinational treatment. As the two drugs have independent modes of mechanism, there will be no conflict between them in targeting different aspects of HCC tumor development, suggesting the potential advantage as a combined treatment rather than a single agent. Sorafenib namely targets Raf-1, B-Raf, VEGFR1–2 and PGDGFR, amongst others, whereas OTX008 inhibits Gal-1 through the binding to a specific location within the CRD to reduce galectin activity [24, 25]. Our results show that the individual treatment of sorafenib and OTX008 significantly reduces tumor growth in the animal model, with this effect also observed in combinational therapy.

## Conclusions

To conclude, this study explores the significance of Gal-1 in promoting HCC tumorigenesis and the potential ways in diminishing its development through shRNA knockdown and introducing miR-22 mimic approaches. Mechanistic analysis revealed the oncogenic capability of Gal-1 in driving HCC cell motility via RER1 upregulation. In vivo experimental models further reinforce the role of Gal-1 in driving HCC tumor growth and metastasis. Furthermore, drug treatment in inhibiting Gal-1 provides promising evidence which could potentially improve patient therapeutic options by reducing the possibility of resistance to one drug and potentially enhance the therapeutic effect of different inhibitors.

## Additional file

Additional file 1: Additional methods, figures and tables. (PDF 414 kb)

## Data Availability

All data generated or analyzed during this study are included in this published article and its supplementary information files.

## Abbreviations

Gal-1: Galectin-1
HBV: Hepatitis B virus
HCC: Hepatocellular carcinoma
miRNA: microRNA
qPCR: Quantitative polymerase chain reaction
RT-qPCR: Reverse transcription-polymerase chain reaction
TCGA: The Cancer Genome Atlas
