# Supplementary material for: Galectin-1 promotes hepatocellular carcinoma and the combined therapeutic effect of OTX008 galectin-1 inhibitor and sorafenib in tumor cells
Source: J Exp Clin Cancer Res. 2019 Oct 22;38:423. doi: 10.1186/s13046-019-1402-x (PMC6805403; doi:10.1186/s13046-019-1402-x)
Supplement: Supplementary file 1 — Additional methods, figures and tables. (PDF 414 kb) [file 13046_2019_1402_MOESM1_ESM.pdf]

Galectin-1 promotes hepatocellular carcinoma and the combined therapeutic effect of OTX008 galectin-1 inhibitor and sorafenib

Zoe Leung[1], Frankie Chi Fat Ko[1], Mao Xiaowen[1], Ernest Man Lok Kwong[1], Bonnie Man Hei Liu[1], Tey Sze Keong[1], Angel Po Yee Ma[1], Yi Man Eva Fung[2,3], Chi-Ming Che[2,3], Danny Ka Ho Wong[4, 5], Ching Lung Lai[4, 5], Irene Oi-Lin Ng[1, 5], Judy Wai Ping Yam[1, 5]

[1]Department of Pathology, The University of Hong Kong, Hong Kong

[2]Department of Chemistry, The University of Hong Kong, Hong Kong

[3]State Key Laboratory of Synthetic Chemistry, The University of Hong Kong, Hong Kong

[4]Department of Medicine, Queen Mary Hospital, The University of Hong Kong, Hong Kong

[5]State Key Laboratory of Liver Research (The University of Hong Kong)

Corresponding author

Judy Wai Ping Yam

Department of Pathology, Block T, Queen Mary Hospital, Pokfulam, Hong Kong.

Tel: (852) 2255-2681; Fax: (852) 2218-5212; E-mail: judyyam@pathology.hku.hk

Supplementary information

Supplementary Methods

Supplementary Figures

Supplementary Table

Supplementary Methods

Antibodies and Taqman probes

The following primary antibodies were selected for the analysis of protein analysis via immunoblot: Galectin-1 (Abcam, Cambridge, UK), HIF1α (Cell Signaling Technologies, Massachusetts, USA), β-actin (Sigma-Aldrich, St Louis, Missouri, USA). The following TaqMan probes were selected and purchased from ThermoFisher Scientific (Waltham, Massachusetts, USA) for RNA analysis by RT-qPCR: LGALS1 (FAM), has-mir-22 (FAM), RNU6 (FAM) and Human HPRT (HGPRT) Endogenous Control (VIC).

Cell lysate extraction and western blot

The successfulness of the stable transfection was analyzed by cell lysate extraction for immunoblot analysis. Cell lysates were obtained by cell lysis with NETN lysis buffer, supplemented with 10% cOmplete™ protease inhibitor cocktail and 10% PhosStop phosphatase inhibitor cocktail (Roche Applied Science, Penzberg, Germany). Total cell lysis was carried out on ice for 30 mins and cleared of cell debris by centrifugation at 4°C. Protein quantification was carried out using Bradford reagent (Bio-Rad Corporation, CA, USA) against a standard concentration of bovine serum albumin (BSA). A total of 30 μg of protein was fractionated on SDS-PAGE, followed by a wet transfer to PVDF membranes (Amersham, GE Healthcare, Buckinghamshire, UK). Membranes were then blocked in 5% non-skimmed milk in TBST. Once sufficiently washed, membranes were incubated in the following primary antibody in their optimized dilutions: Gal-1 (1:2000), β-actin (1:5000), HIF1α (1:1000) and α-tubulin (1:1000). Membranes were incubated overnight at 4°C which were then followed by another wash in TBST before incubation with the appropriate secondary antibody for 1 hour at room temperature, with shaking. Chemiluminescent signals

were then detected using ECL™ Western Blotting Detection Reagents (Amersham™ GE Healthcare).

RNA extraction and Quantitative real time PCR (RT-qPCR)

Total RNA extraction from cells was performed following the TRIzol® reagent protocol (ThermoFisher Scientific, MA, USA). Reverse transcription (RT) for the generation of cDNA was performed with the SuperScript™ VILO™ Master Mix and qPCR with the TaqMan Real-Time PCR Master Mix. Individual samples of each probe were performed in triplicate wells. The data generated from qPCR was analyzed by the ΔΔCT method, with relative Gal-1 and RER1 levels normalized to HPRT and relative miR-22 levels normalized to RNU6. For analyzing Gal-1 expression in HCC tissues, a 2-fold increase and decrease in Gal-1 expression in tumor tissues when compared to non-tumorous tissues was regarded as Gal-1 overexpression and underexpression, respectively.

Plasmid construction and dual-luciferase reporter assay

For the determination of the direct regulation of miR-22 and Gal-1 3'UTR, a luciferase reporter vector was designed as follows: forward primer: 5'-TCGAGATGGCCCC CAATAAAGGCAGCTGCGAATTCGATGGCCCCCAATAAAGAGCT-3'; reverse primer: 5'-CTTTATTGGGGGCCATCGAATTCGCAGCTGCCTTTATTGGGGGCCATC-3'. This set of primers were subjected to sequential temperatures for annealing, first at 95°C for 10 minutes and then reduced to 55°C for 5 minutes before allowing to completely anneal at room temperature for at least 1 hr. This was then followed by T4 PNK (ThermoScientific) treatment for 30 minutes at 37°C. The pmirGLO-Dual luciferase miRNA Target Expression Vector (Promega, Madison, Wisconsin, USA) was cut sequentially with XhoI for 15 minutes

and then with SacI for a further 15 mins. For dephosphorylation, the digested product was treated with Anza™ alkaline phosphatase for 15 mins at 37°C.

The prepared insert and vector were then ligated and successful colonies were selected after transformation into *E.coli* DH5α competent cells. Successful insert clones were purified and used in co-transfection in 293FT. The dual luciferase reporter assay was carried out following the kit's manual (Promega), with the final luciferase activity normalized with the activity of *Renilla* luciferase.

Supplementary Figures

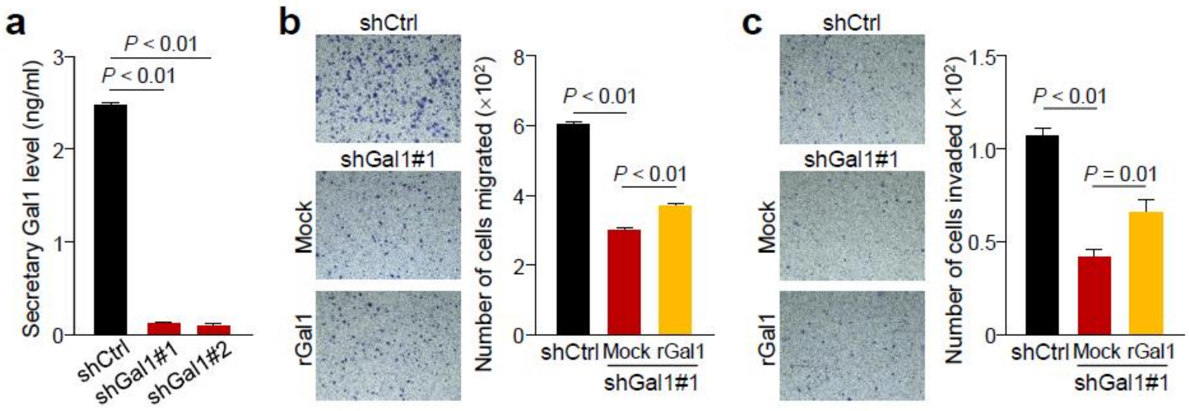

Figure S1. Knockdown of Gal-1 reduced production of secretory Gal-1. a. ELISA analysis revealed the significant reduction in secretory Gal-1 levels in the conditioned medium of control shCtrl and Gal-1 knockdown clones (shGal-1#1 and shGal-1#2) established in MHCC97L cells. Recombinant Gal-1 (rGal1) treatment to MHCC97L shGal1 cells showed that rGal1 was able to enhance cell migration (**b**) and invasion (**c**) despite Gal-1 expression knockdown.

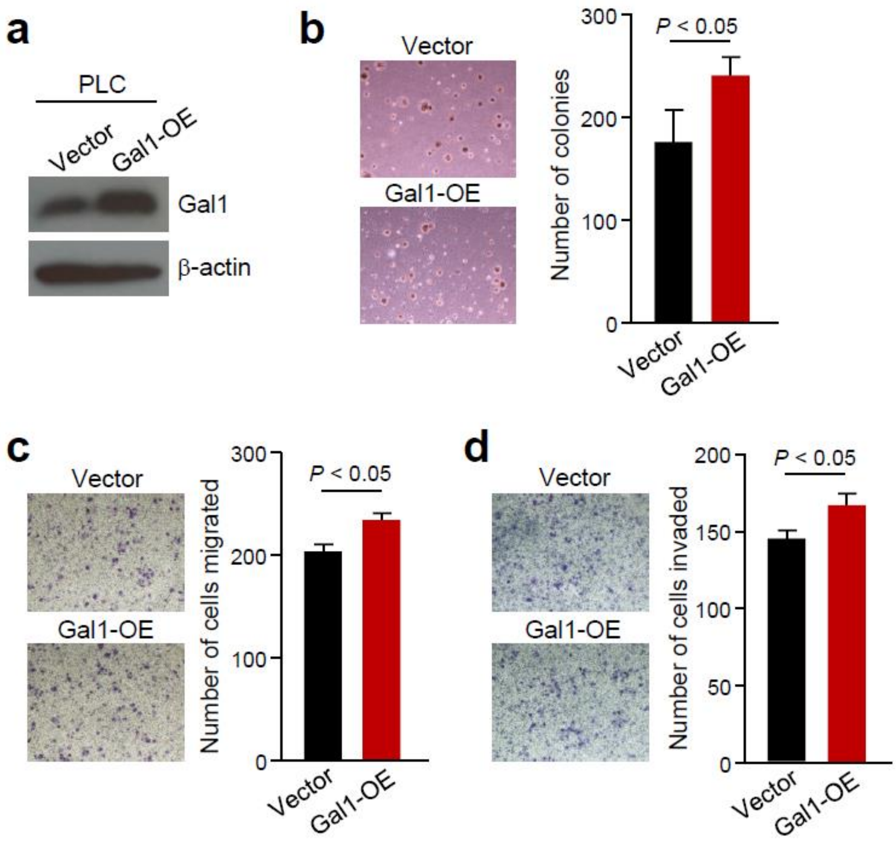

Figure S2. Overexpression of Gal-1 enhanced anchorage independent growth, cell migration and invasiveness in HCC cells. a. Western blotting revealed Gal-1 level in Gal-1 overexpression cells established in PLC/PRF/5. **b.** Significant increase in the number of colonies formed in soft agar assay (**c**). **d.** Gal-1 overexpression in PLC/PRF/5 cells markedly increased cell migration and invasion.

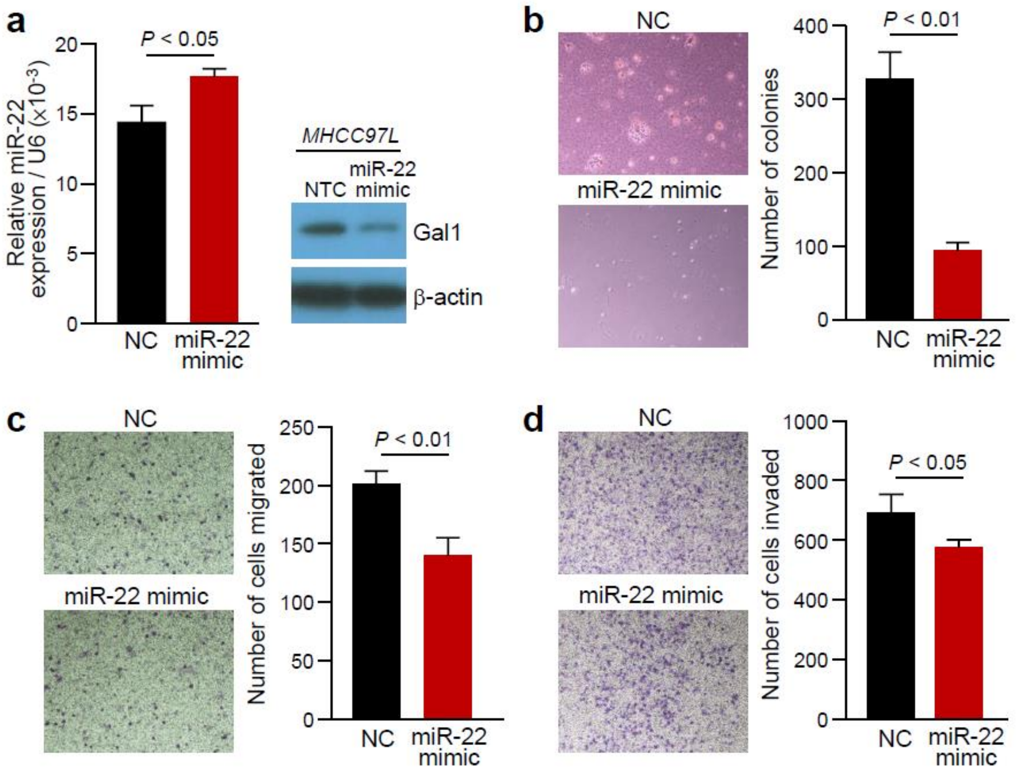

Figure S3. miR-22 negatively regulated HCC cell aggressiveness. a. qPCR revealed enhanced level of miR-22 in stable miR-22 mimic cells when compared to the non-target control (NC) cells established in MHCC97L. Western blotting revealed the concomitant downregulation of Gal-1 level in stable miR-22 mimic cells. **b.** Significant decrease in the number of colonies formed in soft agar assay. Migration (**c**) and invasion (**d**) assays revealed diminished migratory and invasive potentials of stable miR-22 mimic cells.

Table S1: Clinicopathologial analysis of miR-22 in HCC clinical samples

Histopathological parameters		T/NT ≤ 0.5 (with miR22 underexpression)	T/NT > 0.5 (without miR22 underexpression)	*P*-value
Sex	Male	26	29	0.609
	Female	11	9	
Cirrhotic liver	Cirrhosis	7	10	0.542
	NT & hepatitis	15	13	
HBsAg	Positive	19	23	1.000
	Negative	3	4	
Cellular differentiation	Poor	7	14	0.075
	Differentiated	15	9	
Tumour size	>5 cm	15	15	1.000
	≤5 cm	7	8	
Tumour encapsulation	Absent	12	15	0.587
	Present	14	12	
Venous invasion	Present	15	21	0.098
	Absent	22	13	
Microsatellite	Present	7	15	0.049
	Absent	16	10	
Direct liver invasion	Present	7	6	0.520
	Absent	11	15	
Tumour nodule	N ≥ 2	5	5	1.000
	N = 1	17	17	

T, Tumorous; NT, non-tumourous; N, number of tumor nodule; HBsAg, Hepatitis B surface antigen; $P < 0.05$ is regarded as statistically significant
